# Supplementary material for: Long‐Term Ecological Baselines and Critical Thresholds in Ombrotrophic Peatlands of Europe: Implications for Restoration Strategies
Source: Glob Chang Biol. 2025 Dec 11;31(12):e70629. doi: 10.1111/gcb.70629 (PMC12696514; doi:10.1111/gcb.70629)
Supplement: Supplementary file 1 — Data S1: gcb70629‐sup‐0001‐FigureS1‐S21‐TableS1.pdf. [file GCB-31-e70629-s001.pdf]

Supplementary material captions

Long-Term Ecological Baselines and Critical Thresholds in Ombrotrophic Peatlands of Europe: Implications for Restoration Strategies

Supplementary Material

Supplementary Figures

- **Supplementary Figure 1** — Bayesian age-depth model (based on ^{14}C dating), *Amtsvenn-Hündfelder Moor*
- **Supplementary Figure 2** — Bayesian age-depth model (based on ^{14}C dating), *Bagno Kusowo*
- **Supplementary Figure 3** — Bayesian age-depth model (based on ^{14}C dating), *Drebbersches Moor*
- **Supplementary Figure 4** — Bayesian age-depth model (based on ^{14}C dating), *Fochteloer Veen*
- **Supplementary Figure 5** — Bayesian age-depth model (based on ^{14}C dating), *Pichlmeier Moor*
- **Supplementary Figure 6** — Bayesian age-depth model (based on ^{14}C dating), *Pürgschachen Moor*
- **Supplementary Figure 7** — Bayesian age-depth model (based on ^{14}C dating), *Store Mosse*
- **Supplementary Figure 8** — Plant macrofossils diagram, *Amtsvenn-Hündfelder Moor*
- **Supplementary Figure 9** — Plant macrofossils diagram, *Bagno Kusowo*
- **Supplementary Figure 10** — Plant macrofossils diagram, *Drebbersches Moor*
- **Supplementary Figure 11** — Plant macrofossils diagram, *Fochteloer Veen*

- **Supplementary Figure 12** — Plant macrofossils diagram, *Pichlmeier Moor*
- **Supplementary Figure 13** — Plant macrofossils diagram, *Pürgschachen Moor*
- **Supplementary Figure 14** — Plant macrofossils diagram, *Store Mosse*
- **Supplementary Figure 15** — Percentage testate amoebae diagram, *Amtsvenn-Hündfelder Moor*
- **Supplementary Figure 16** — Percentage testate amoebae diagram, *Bagno Kusowo*
- **Supplementary Figure 17** — Percentage testate amoebae diagram, *Drebbersches Moor*
- **Supplementary Figure 18** — Percentage testate amoebae diagram, *Fochteloer Veen*
- **Supplementary Figure 19** — Percentage testate amoebae diagram, *Pichlmeier Moor*
- **Supplementary Figure 20** — Percentage testate amoebae diagram, *Pürgschachen Moor*
- **Supplementary Figure 21** — Percentage testate amoebae diagram, *Store Mosse*

Supplementary Tables

- **Supplementary Table 1** — The list of radiocarbon dates from seven studied peatlands with calibration. The IntCal20 (Reimer et al., 2020) and Bomb21NH1 (Hua et al., 2021) atmospheric curves were used to calibrate the dates. *pMC*– percent modern carbon.

Suppl. Fig. 1

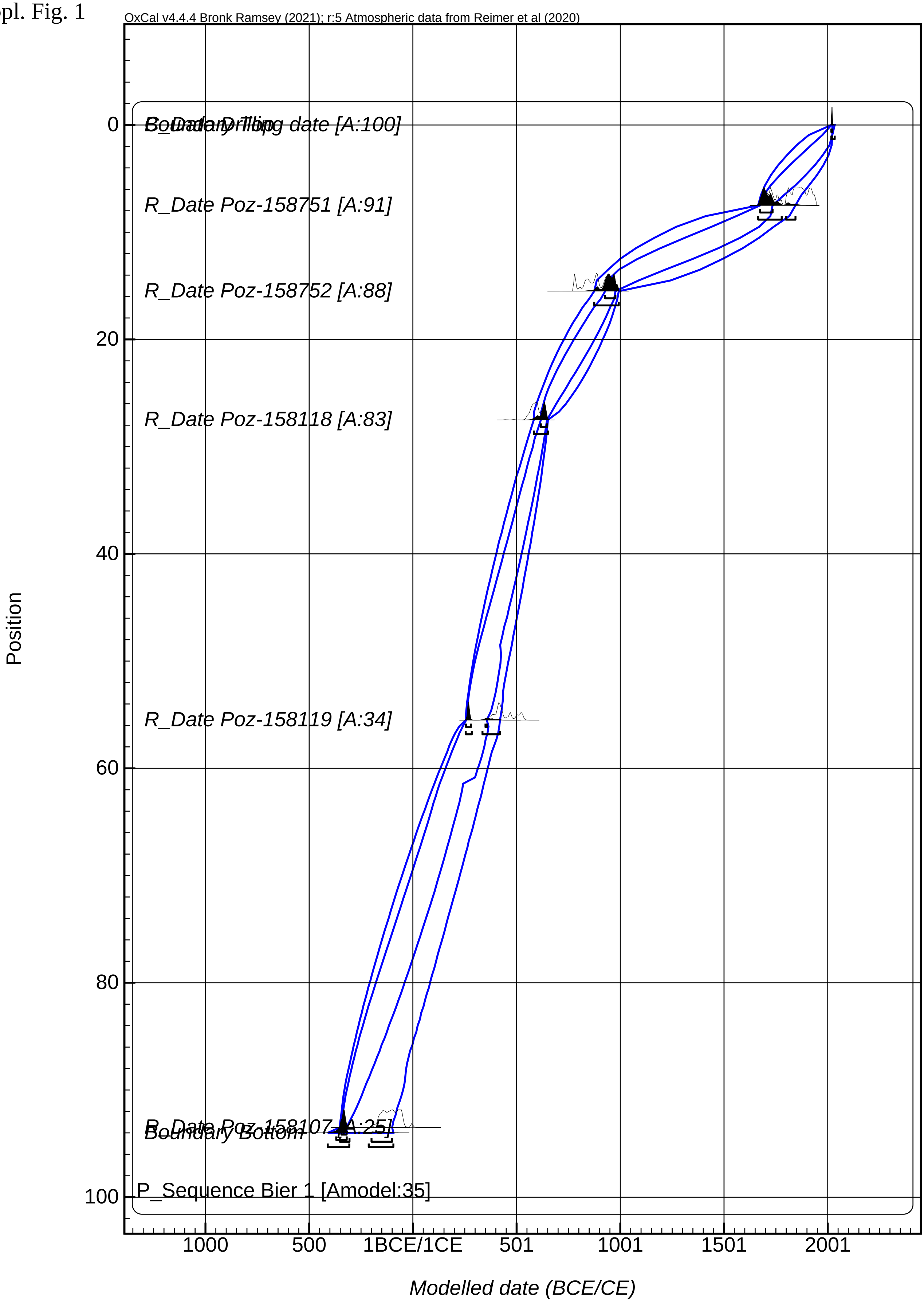
OxCal v4.4.4 Bronk Ramsey (2021); r:5 Atmospheric data from Reimer et al (2020)
Boundary Top
C_Date Drilling date [A:100]
R_Date Poz-158751 [A:91]
R_Date Poz-158752 [A:88]
R_Date Poz-158118 [A:83]
R_Date Poz-158119 [A:34]
R_Date Poz-158107 [A:25]
Boundary Bottom
P_Sequence Bier 1 [Amodel:35]
0
20
40
60
80
100
Position
1000
500
1BCE/1CE
501
1001
1501
2001
Modelled date (BCE/CE)

Suppl. Fig. 2

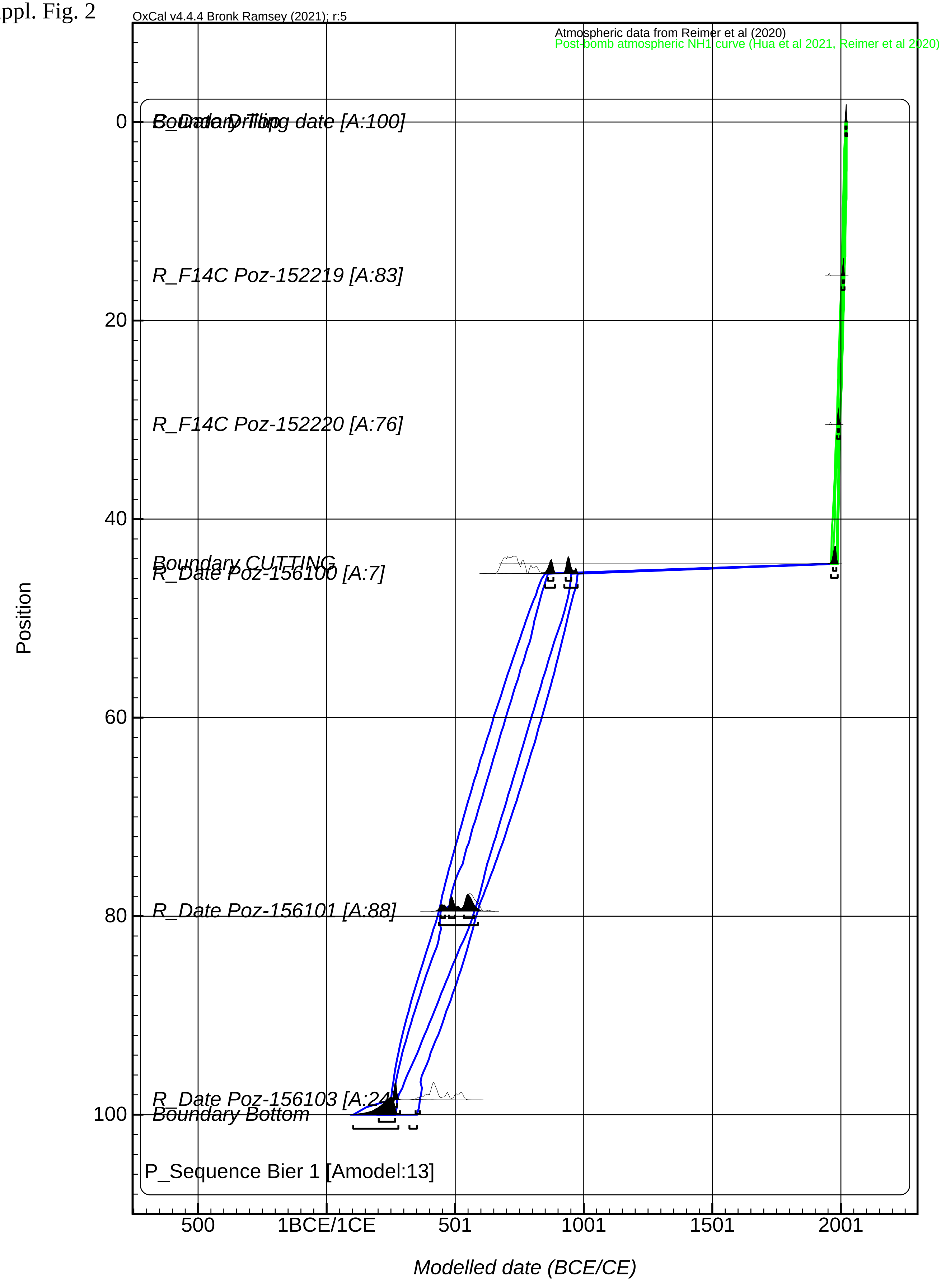
OxCal v4.4.4 Bronk Ramsey (2021); r:5
Atmospheric data from Reimer et al (2020)
Post-bomb atmospheric NH1 curve (Hua et al 2021, Reimer et al 2020)
Boundary Top
C_Date Drilling date [A:100]
R_F14C Poz-152219 [A:83]
R_F14C Poz-152220 [A:76]
Boundary CUTTING
R_Date Poz-156100 [A:7]
R_Date Poz-156101 [A:88]
R_Date Poz-156103 [A:24]
Boundary Bottom
P_Sequence Bier 1 [Amodel:13]
Position
0
20
40
60
80
100
500
1BCE/1CE
501
1001
1501
2001
Modelled date (BCE/CE)

Suppl. Fig. 3

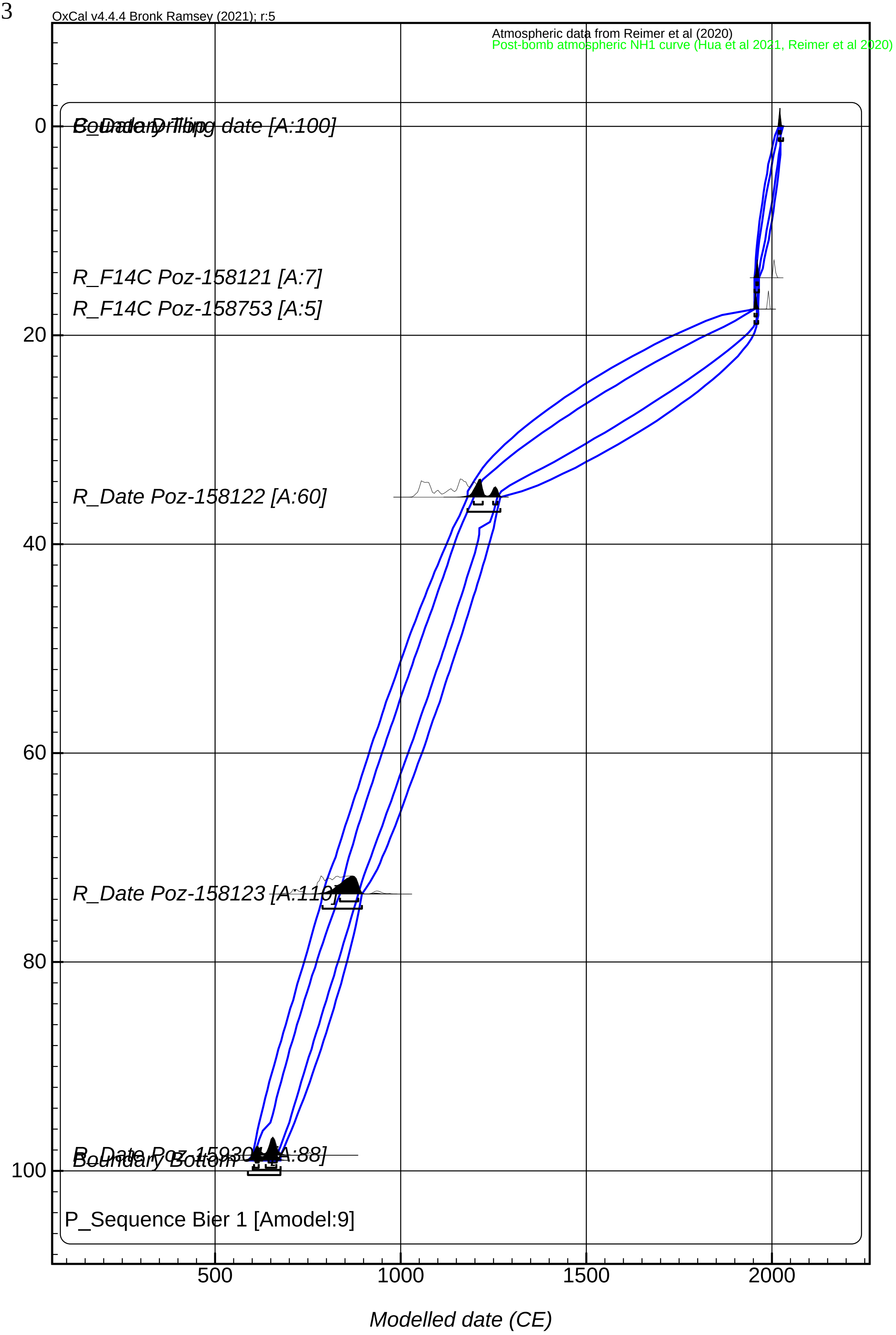

OxCal v4.4.4 Bronk Ramsey (2021); r:5
Atmospheric data from Reimer et al (2020)
Post-bomb atmospheric NH1 curve (Hua et al 2021, Reimer et al 2020)
Boundary Top
C_Date Drilling date [A:100]
R_F14C Poz-158121 [A:7]
R_F14C Poz-158753 [A:5]
R_Date Poz-158122 [A:60]
R_Date Poz-158123 [A:110]
R_Date Poz-159301 [A:88]
Boundary Bottom
P_Sequence Bier 1 [Amodel:9]
0
20
40
60
80
100
Position
500
1000
1500
2000
Modelled date (CE)

Suppl. Fig. 4

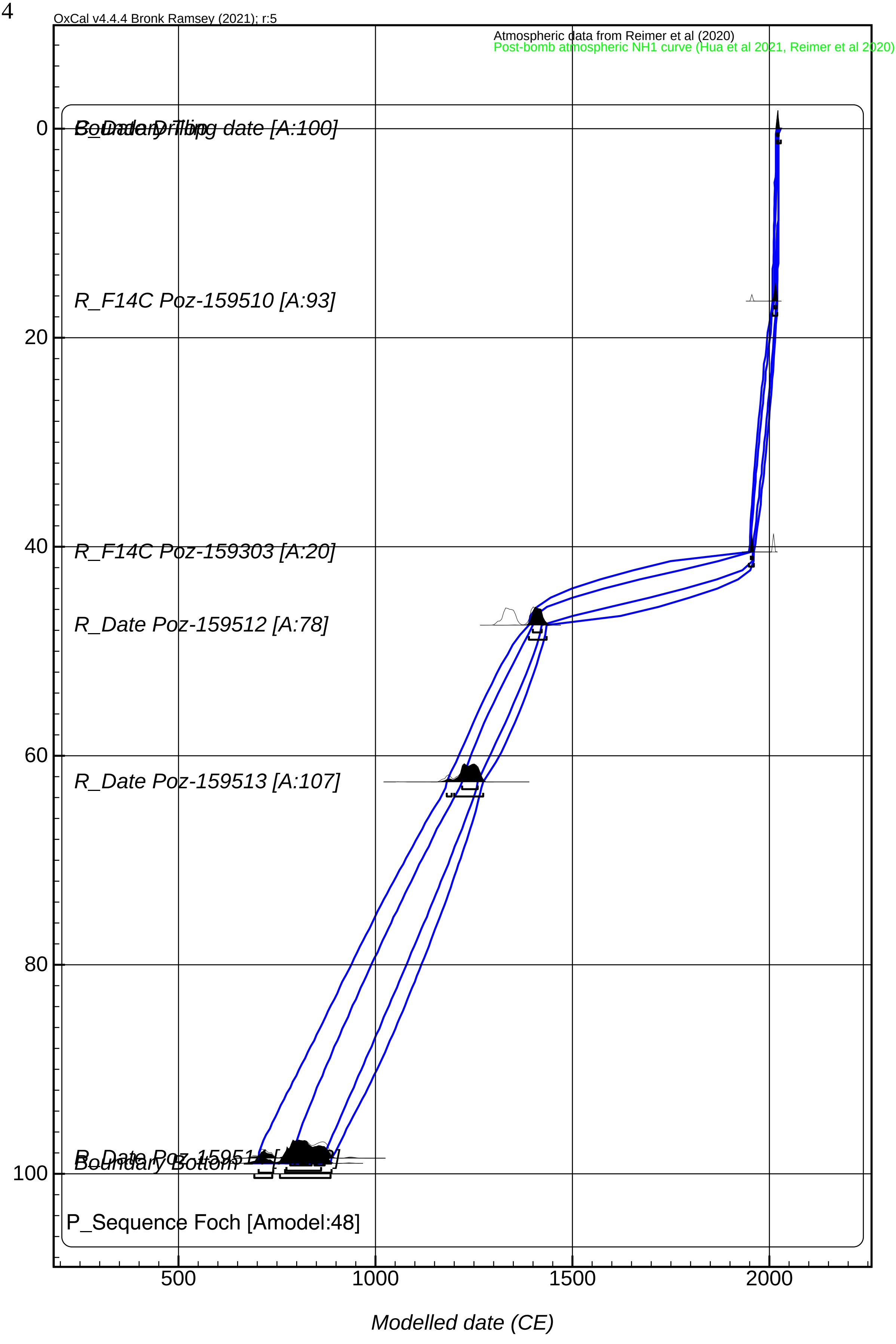
OxCal v4.4.4 Bronk Ramsey (2021); r:5
Atmospheric data from Reimer et al (2020)
Post-bomb atmospheric NH1 curve (Hua et al 2021, Reimer et al 2020)
Boundary Top
C_Date Drilling date [A:100]
R_F14C Poz-159510 [A:93]
R_F14C Poz-159303 [A:20]
R_Date Poz-159512 [A:78]
R_Date Poz-159513 [A:107]
Boundary Bottom
P_Sequence Foch [Amodel:48]
0
20
40
60
80
100
Position
500
1000
1500
2000
Modelled date (CE)

Suppl. Fig. 5

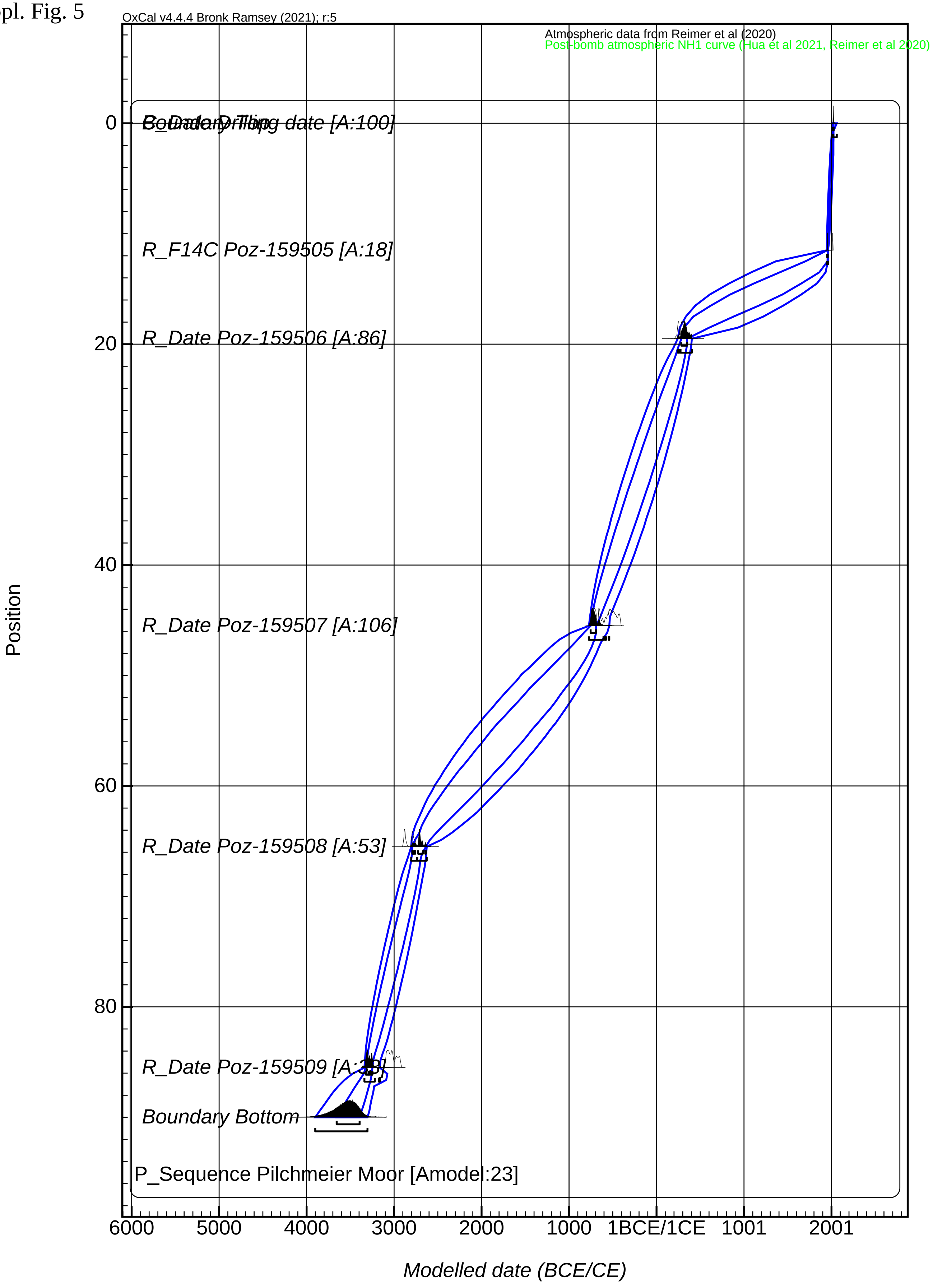
OxCal v4.4.4 Bronk Ramsey (2021); r:5
Atmospheric data from Reimer et al (2020)
Post-bomb atmospheric NH1 curve (Hua et al 2021, Reimer et al 2020)
Boundary Top
C_Date Drilling date [A:100]
R_F14C Poz-159505 [A:18]
R_Date Poz-159506 [A:86]
R_Date Poz-159507 [A:106]
R_Date Poz-159508 [A:53]
R_Date Poz-159509 [A:33]
Boundary Bottom
P_Sequence Pilchmeier Moor [Amodel:23]
Position
0
20
40
60
80
6000
5000
4000
3000
2000
1000
1BCE/1CE
1001
2001
Modelled date (BCE/CE)

Suppl. Fig. 6

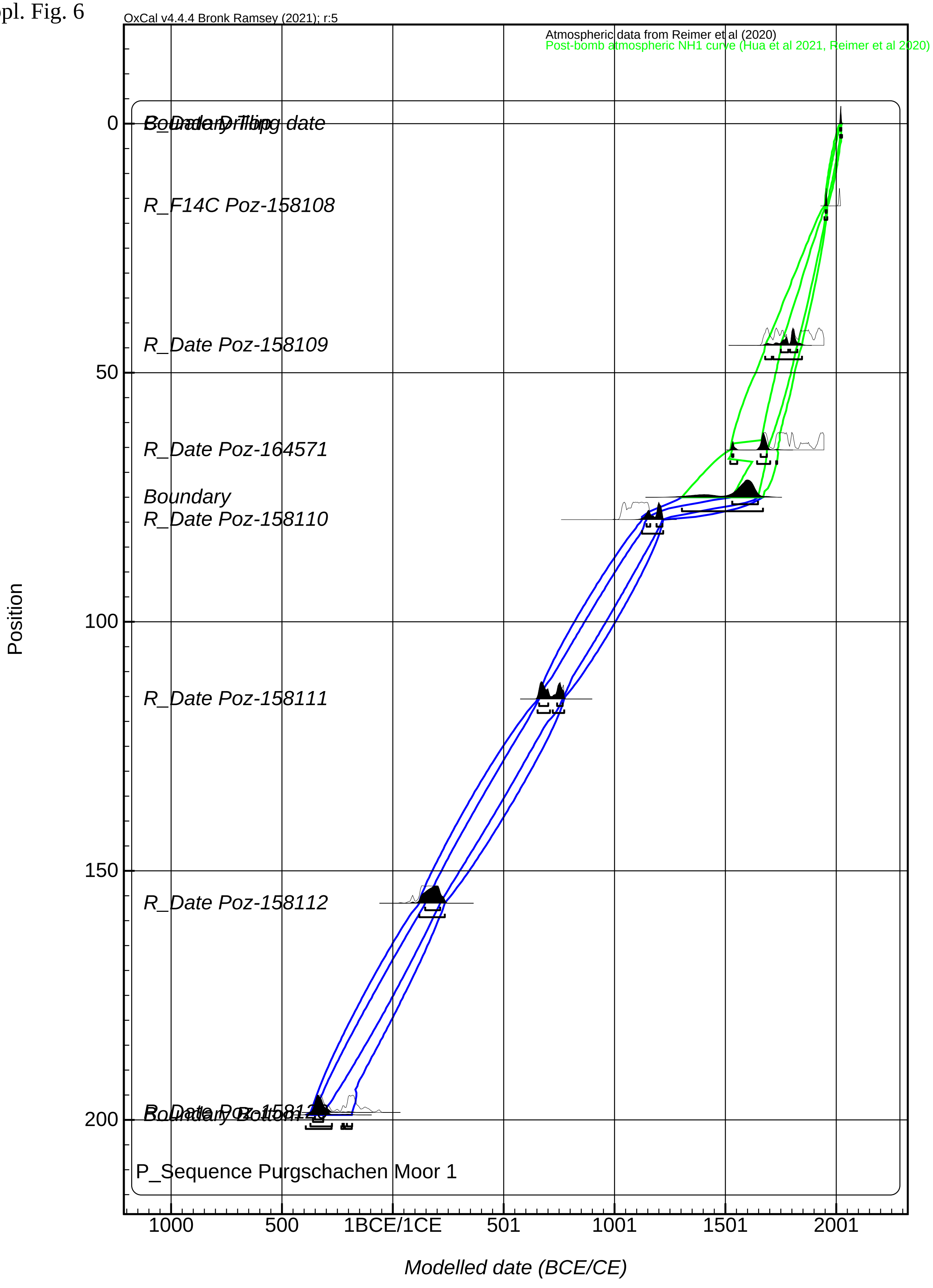

OxCal v4.4.4 Bronk Ramsey (2021); r:5
Atmospheric data from Reimer et al (2020)
Post-bomb atmospheric NH1 curve (Hua et al 2021, Reimer et al 2020)
Boundary Top
C_Date Drilling date
R_F14C Poz-158108
R_Date Poz-158109
R_Date Poz-164571
Boundary
R_Date Poz-158110
R_Date Poz-158111
R_Date Poz-158112
R_Date Poz-158113
Boundary Bottom
P_Sequence Purgschachen Moor 1
0
50
100
150
200
1000
500
1BCE/1CE
501
1001
1501
2001
Position
Modelled date (BCE/CE)

Suppl. Fig. 7

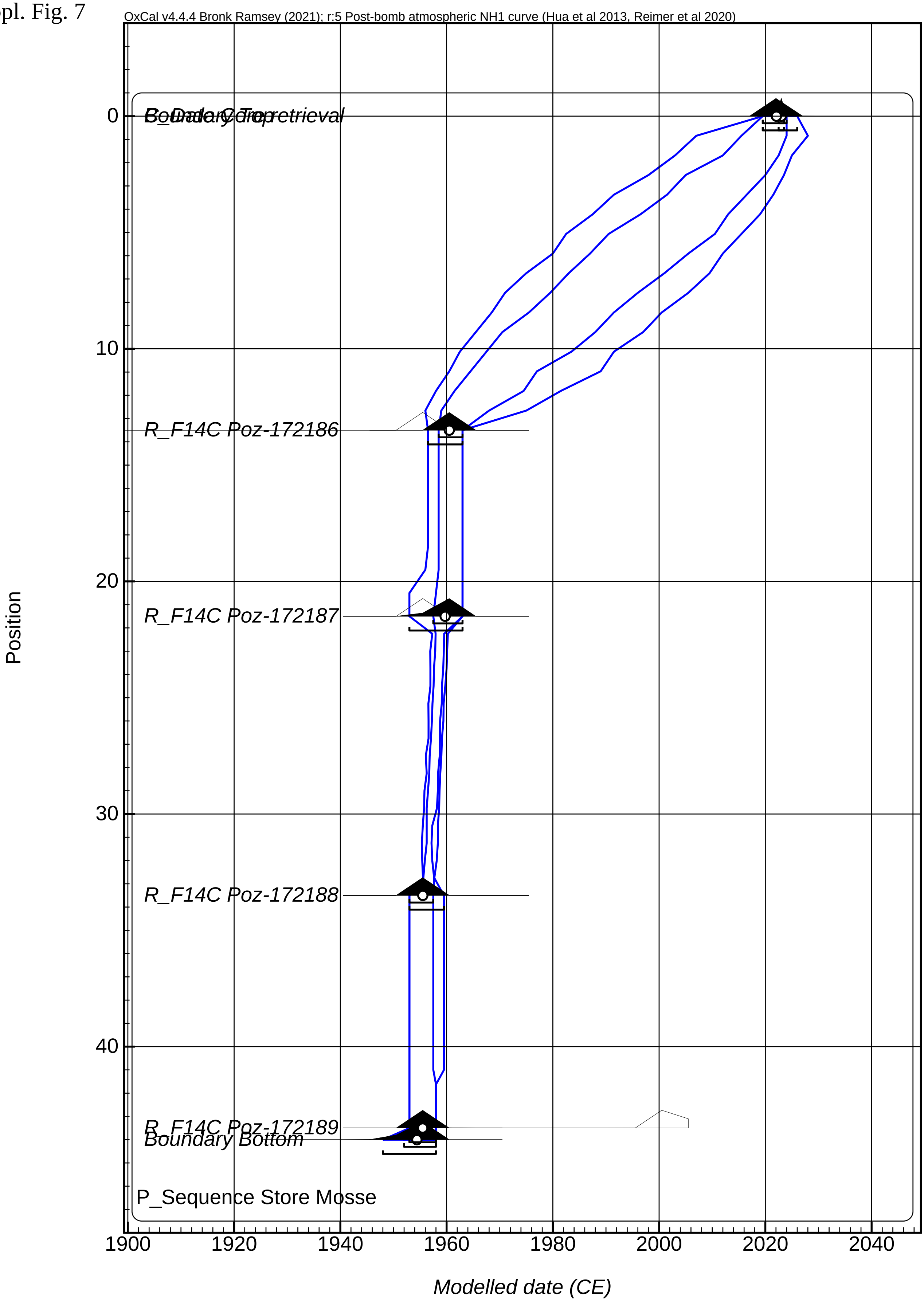
OxCal v4.4.4 Bronk Ramsey (2021); r:5 Post-bomb atmospheric NH1 curve (Hua et al 2013, Reimer et al 2020)
Boundary Top
C_Date Core retrieval
R_F14C Poz-172186
R_F14C Poz-172187
R_F14C Poz-172188
R_F14C Poz-172189
Boundary Bottom
P_Sequence Store Mosse
0
10
20
30
40
Position
1900
1920
1940
1960
1980
2000
2020
2040
Modelled date (CE)

Suppl. Fig. 8

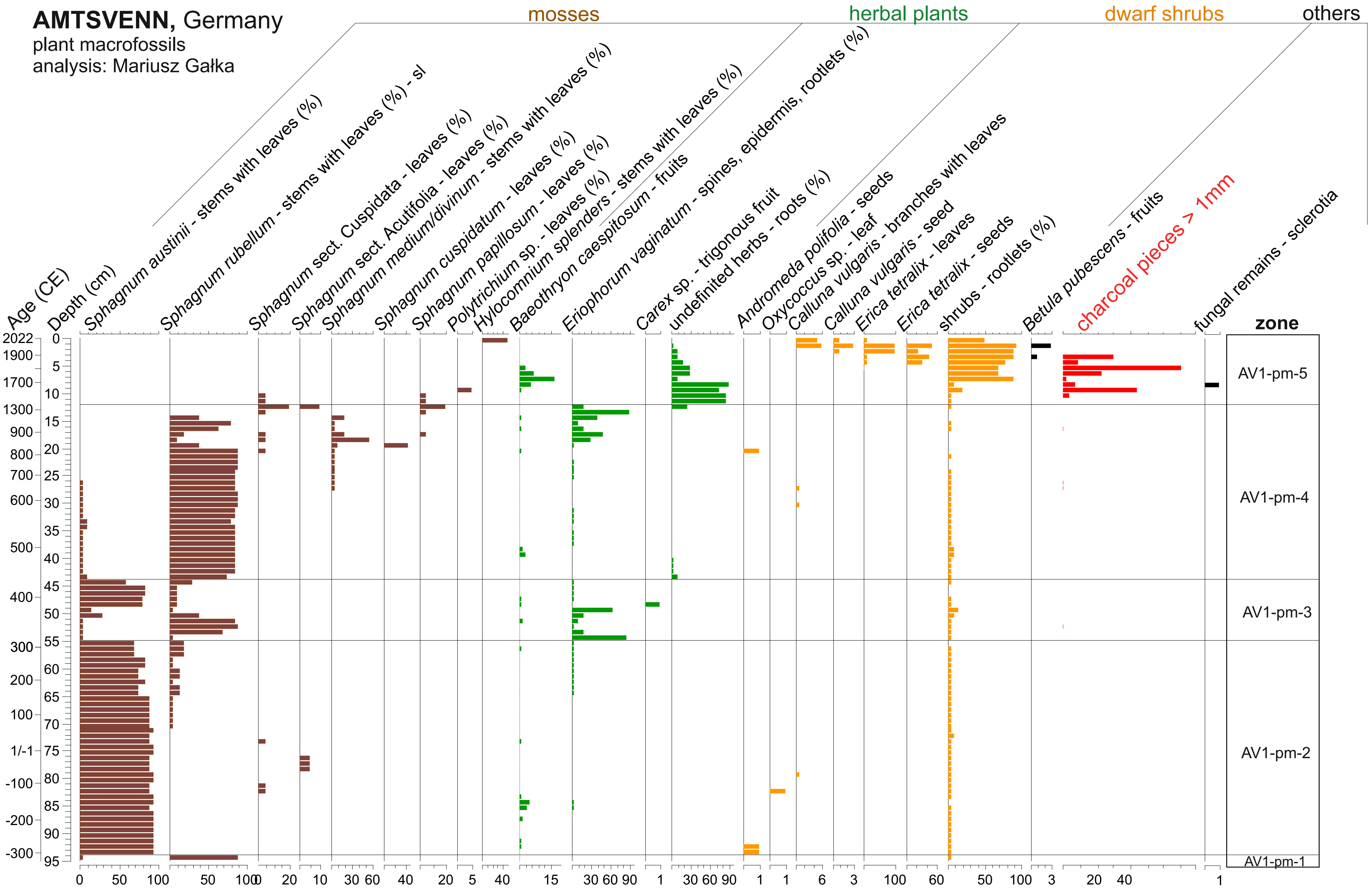

AMTSVENN, Germany
plant macrofossils
analysis: Mariusz Gałka
mosses
herbal plants
dwarf shrubs
others
Age (CE)
Depth (cm)
Sphagnum austinii - stems with leaves (%)
Sphagnum rubellum - stems with leaves (%) - sl
Sphagnum sect. Cuspidata - leaves (%)
Sphagnum sect. Acutifolia - leaves (%)
Sphagnum medium/divinum - stems with leaves (%)
Sphagnum cuspidatum - leaves (%)
Sphagnum papillosum - leaves (%)
Polytrichium sp. - leaves (%)
Hylocomnium splenders - stems with leaves (%)
Baeothryon caespitosum - fruits
Eriophorum vaginatum - spines, epidermis, rootlets (%)
Carex sp. - trigonous fruit
undefinited herbs - roots (%)
Andromeda polifolia - seeds
Oxycoccus sp. - leaf
Calluna vulgaris - branches with leaves
Calluna vulgaris - seed
Erica tetralix - leaves
Erica tetralix - seeds
shrubs - rootlets (%)
Betula pubescens - fruits
charcoal pieces > 1mm
fungal remains - sclerotia
zone
AV1-pm-5
AV1-pm-4
AV1-pm-3
AV1-pm-2
AV1-pm-1

Suppl. Fig. 9

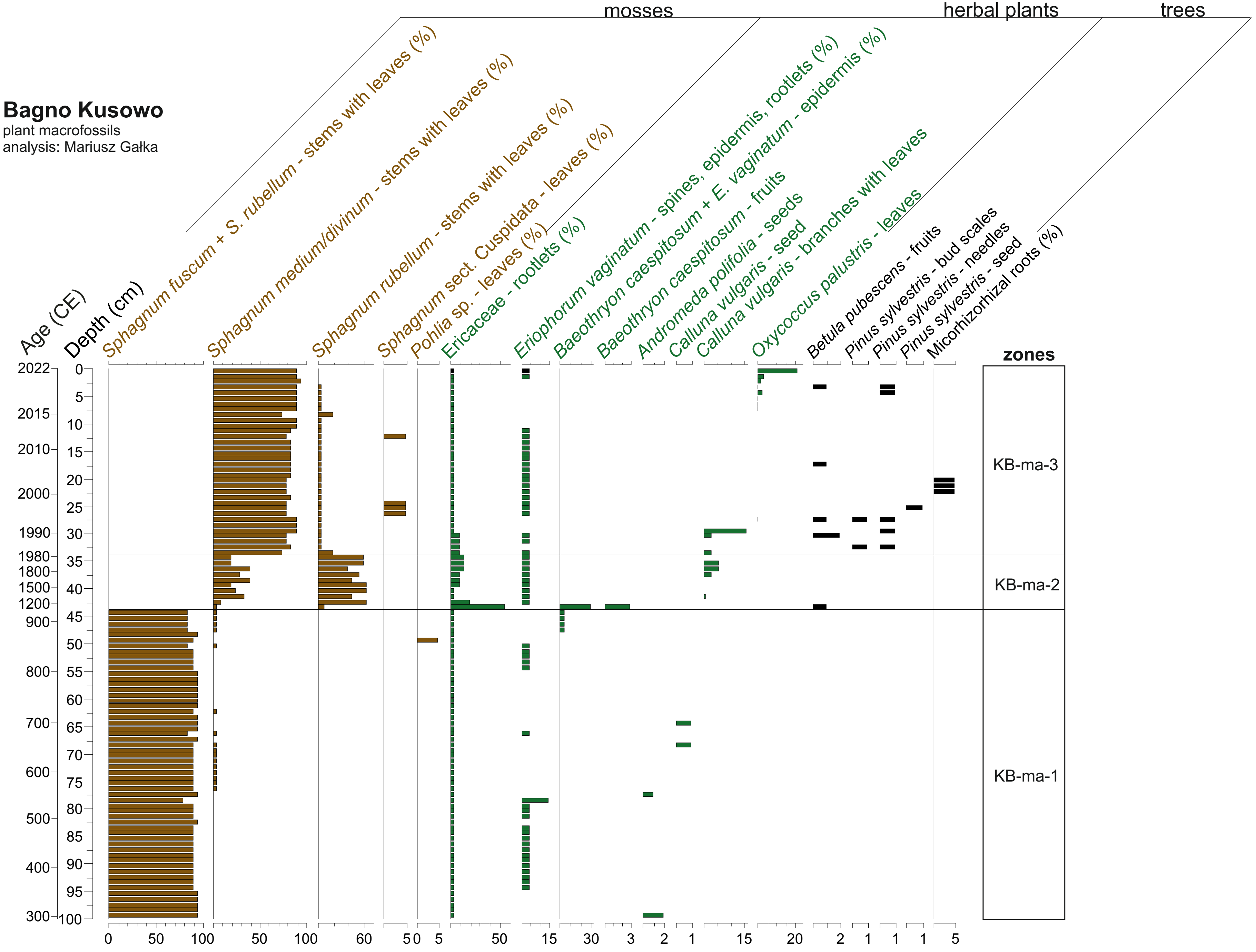
Bagno Kusowo
plant macrofossils
analysis: Mariusz Gałka
mosses
herbal plants
trees
Age (CE)
Depth (cm)
Sphagnum fuscum + S. rubellum - stems with leaves (%)
Sphagnum medium/divinum - stems with leaves (%)
Sphagnum rubellum - stems with leaves (%)
Sphagnum sect. Cuspidata - leaves (%)
Pohlia sp. - leaves (%)
Ericaceae - rootlets (%)
Eriophorum vaginatum - spines, epidermis, rootlets (%)
Baeothryon caespitosum + E. vaginatum - epidermis (%)
Baeothryon caespitosum - fruits
Andromeda polifolia - seeds
Calluna vulgaris - seed
Calluna vulgaris - branches with leaves
Oxycoccus palustris - leaves
Betula pubescens - fruits
Pinus sylvestris - bud scales
Pinus sylvestris - needles
Pinus sylvestris - seed
Micorhizorhizal roots (%)
zones
KB-ma-3
KB-ma-2
KB-ma-1

Suppl. Fig. 10

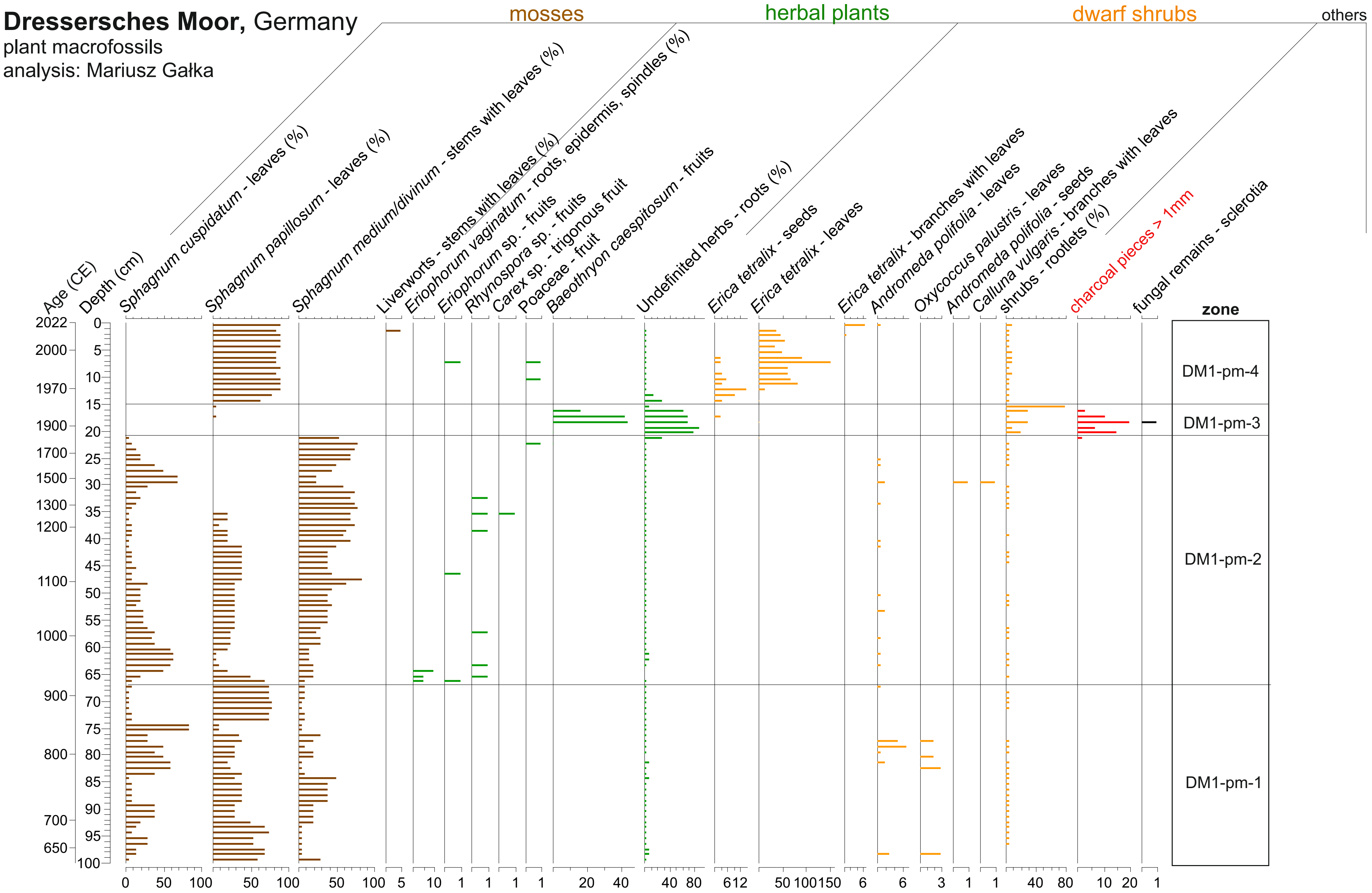

Dressersches Moor, Germany
plant macrofossils
analysis: Mariusz Gałka
mosses
herbal plants
dwarf shrubs
others
Age (CE)
Depth (cm)
Sphagnum cuspidatum - leaves (%)
Sphagnum papillosum - leaves (%)
Sphagnum medium/divinum - stems with leaves (%)
Liverworts - stems with leaves (%)
Eriophorum vaginatum - roots, epidermis, spindles (%)
Eriophorum sp. - fruits
Rhynospora sp. - fruits
Carex sp. - trigonous fruit
Poaceae - fruit
Baeothryon caespitosum - fruits
Undefinited herbs - roots (%)
Erica tetralix - seeds
Erica tetralix - leaves
Erica tetralix - branches with leaves
Andromeda polifolia - leaves
Oxycoccus palustris - leaves
Andromeda polifolia - seeds
Calluna vulgaris - branches with leaves
shrubs - rootlets (%)
charcoal pieces > 1mm
fungal remains - sclerotia
zone
DM1-pm-4
DM1-pm-3
DM1-pm-2
DM1-pm-1

Suppl. Fig. 11

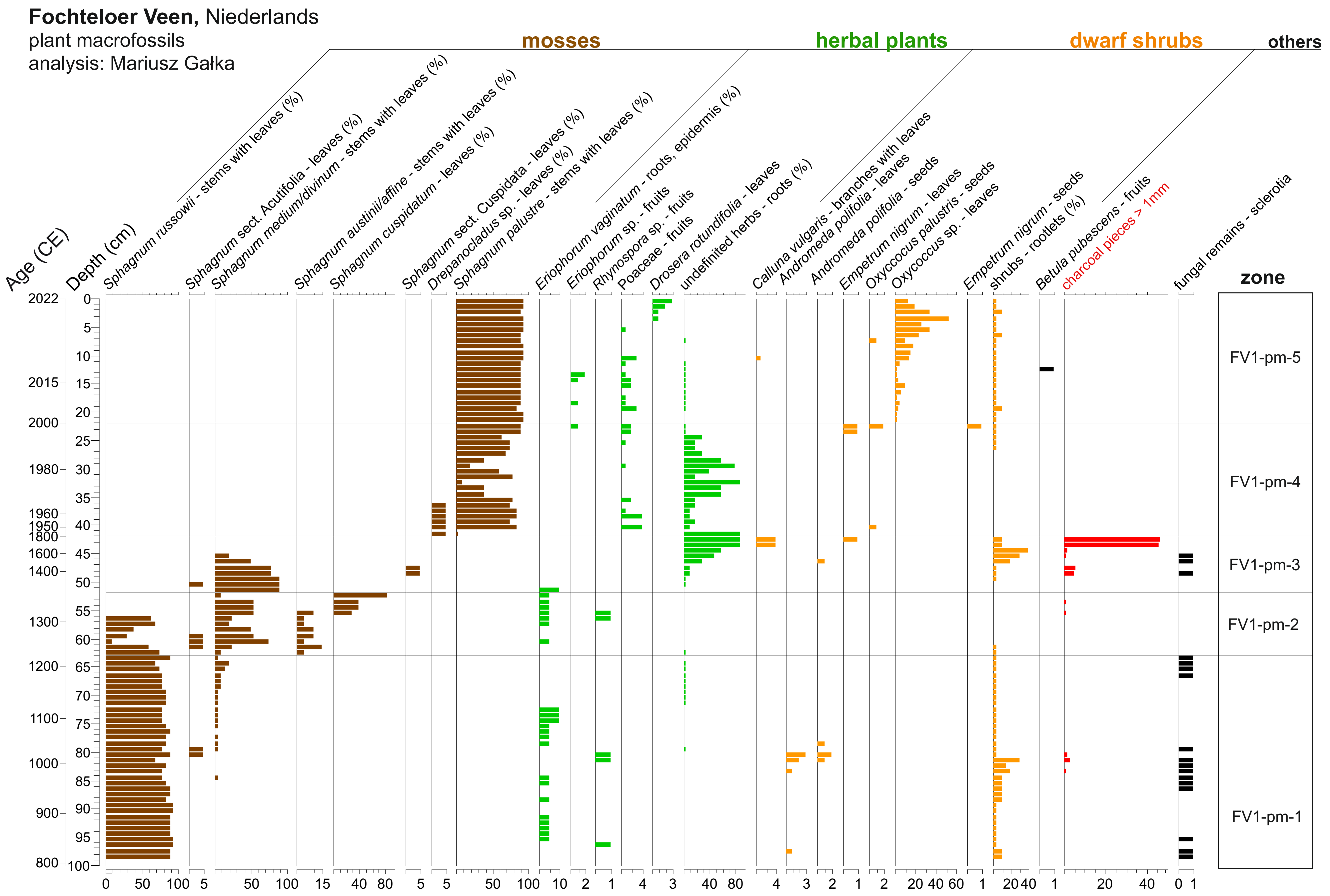

Fochteloer Veen, Niederlands
plant macrofossils
analysis: Mariusz Gałka
mosses
herbal plants
dwarf shrubs
others
Age (CE)
Depth (cm)
Sphagnum russowii - stems with leaves (%)
Sphagnum sect. Acutifolia - leaves (%)
Sphagnum medium/divinum - stems with leaves (%)
Sphagnum austinii/affine - stems with leaves (%)
Sphagnum cuspidatum - leaves (%)
Sphagnum sect. Cuspidata - leaves (%)
Drepanocladus sp. - leaves (%)
Sphagnum palustre - stems with leaves (%)
Eriophorum vaginatum - roots, epidermis (%)
Eriophorum sp. - fruits
Rhynospora sp. - fruits
Poaceae - fruits
Drosera rotundifolia - leaves
undefinited herbs - roots (%)
Calluna vulgaris - branches with leaves
Andromeda polifolia - leaves
Andromeda polifolia - seeds
Empetrum nigrum - leaves
Oxyccocus palustris - seeds
Oxyccocus sp. - leaves
Empetrum nigrum - seeds
shrubs - rootlets (%)
Betula pubescens - fruits
charcoal pieces > 1mm
fungal remains - sclerotia
zone
FV1-pm-5
FV1-pm-4
FV1-pm-3
FV1-pm-2
FV1-pm-1

Suppl. Fig. 12

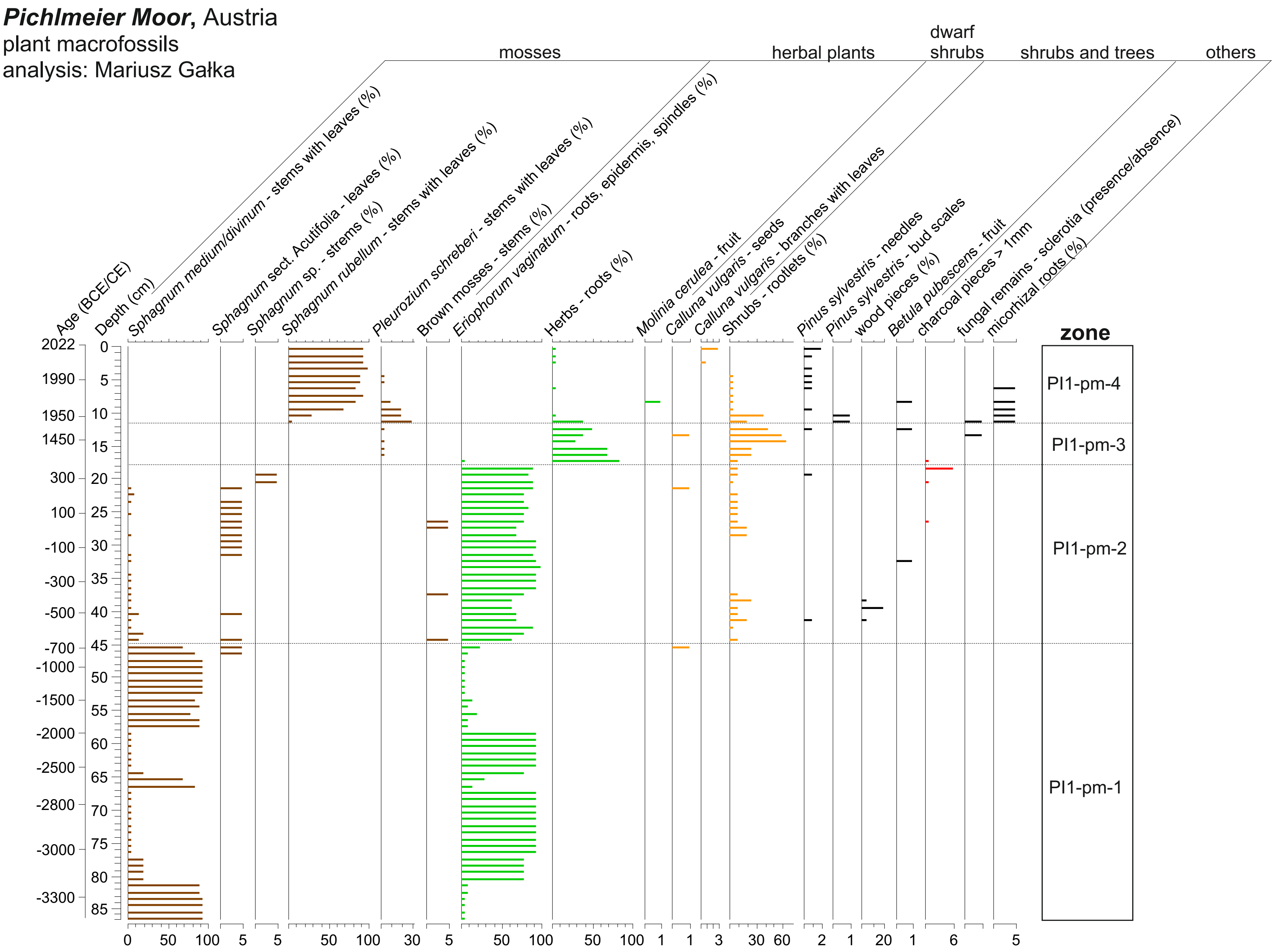

Pichlmeier Moor, Austria
plant macrofossils
analysis: Mariusz Gałka
mosses
herbal plants
dwarf shrubs
shrubs and trees
others
Age (BCE/CE)
Depth (cm)
Sphagnum medium/divinum - stems with leaves (%)
Sphagnum sect. Acutifolia - leaves (%)
Sphagnum sp. - strems (%)
Sphagnum rubellum - stems with leaves (%)
Pleurozium schreberi - stems with leaves (%)
Brown mosses - stems (%)
Eriophorum vaginatum - roots, epidermis, spindles (%)
Herbs - roots (%)
Molinia cerulea - fruit
Calluna vulgaris - seeds
Calluna vulgaris - branches with leaves
Shrubs - rootlets (%)
Pinus sylvestris - needles
Pinus sylvestris - bud scales
wood pieces (%)
Betula pubescens - fruit
charcoal pieces > 1mm
fungal remains - sclerotia (presence/absence)
micorhizal roots (%)
zone
PI1-pm-4
PI1-pm-3
PI1-pm-2
PI1-pm-1
2022
1990
1950
1450
300
100
-100
-300
-500
-700
-1000
-1500
-2000
-2500
-2800
-3000
-3300
0
5
10
15
20
25
30
35
40
45
50
55
60
65
70
75
80
85
0 50 100
5
5
50 100
30
5
50 100
50 100
1
1
3
30 60
2
1
20
1
6
5

Suppl. Fig. 13

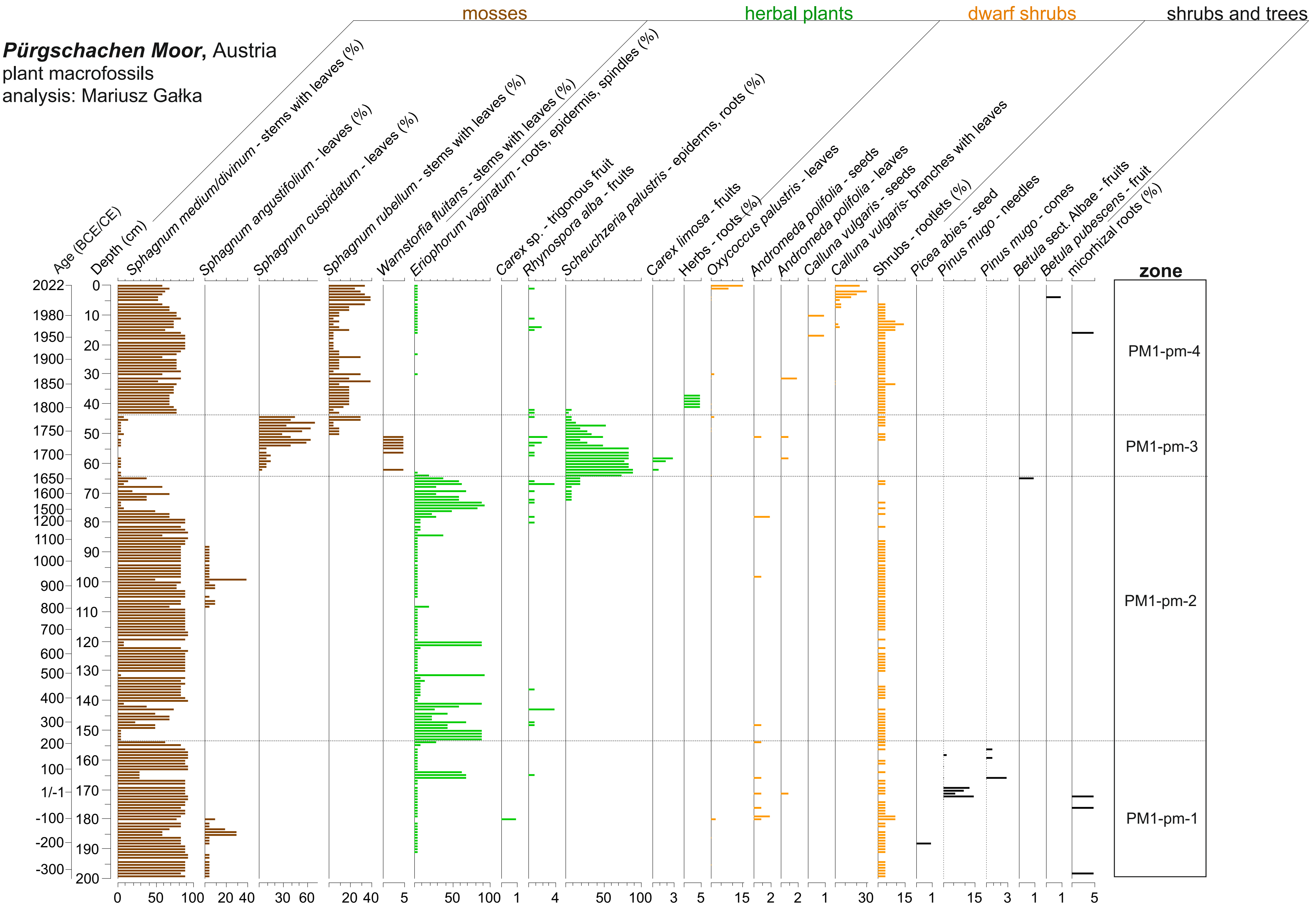

Pürgschachen Moor, Austria
plant macrofossils
analysis: Mariusz Gałka
mosses
herbal plants
dwarf shrubs
shrubs and trees
Age (BCE/CE)
Depth (cm)
Sphagnum medium/divinum - stems with leaves (%)
Sphagnum angustifolium - leaves (%)
Sphagnum cuspidatum - leaves (%)
Sphagnum rubellum - stems with leaves (%)
Warnstorfia fluitans - stems with leaves (%)
Eriophorum vaginatum - roots, epidermis, spindles (%)
Carex sp. - trigonous fruit
Rhynospora alba - fruits
Scheuchzeria palustris - epiderms, roots (%)
Carex limosa - fruits
Herbs - roots (%)
Oxycoccus palustris - leaves
Andromeda polifolia - seeds
Andromeda polifolia - leaves
Calluna vulgaris - seeds
Calluna vulgaris- branches with leaves
Shrubs - rootlets (%)
Picea abies - seed
Pinus mugo - needles
Pinus mugo - cones
Betula sect. Albae - fruits
Betula pubescens - fruit
micorhizal roots (%)
zone
PM1-pm-4
PM1-pm-3
PM1-pm-2
PM1-pm-1

Suppl. Fig. 14

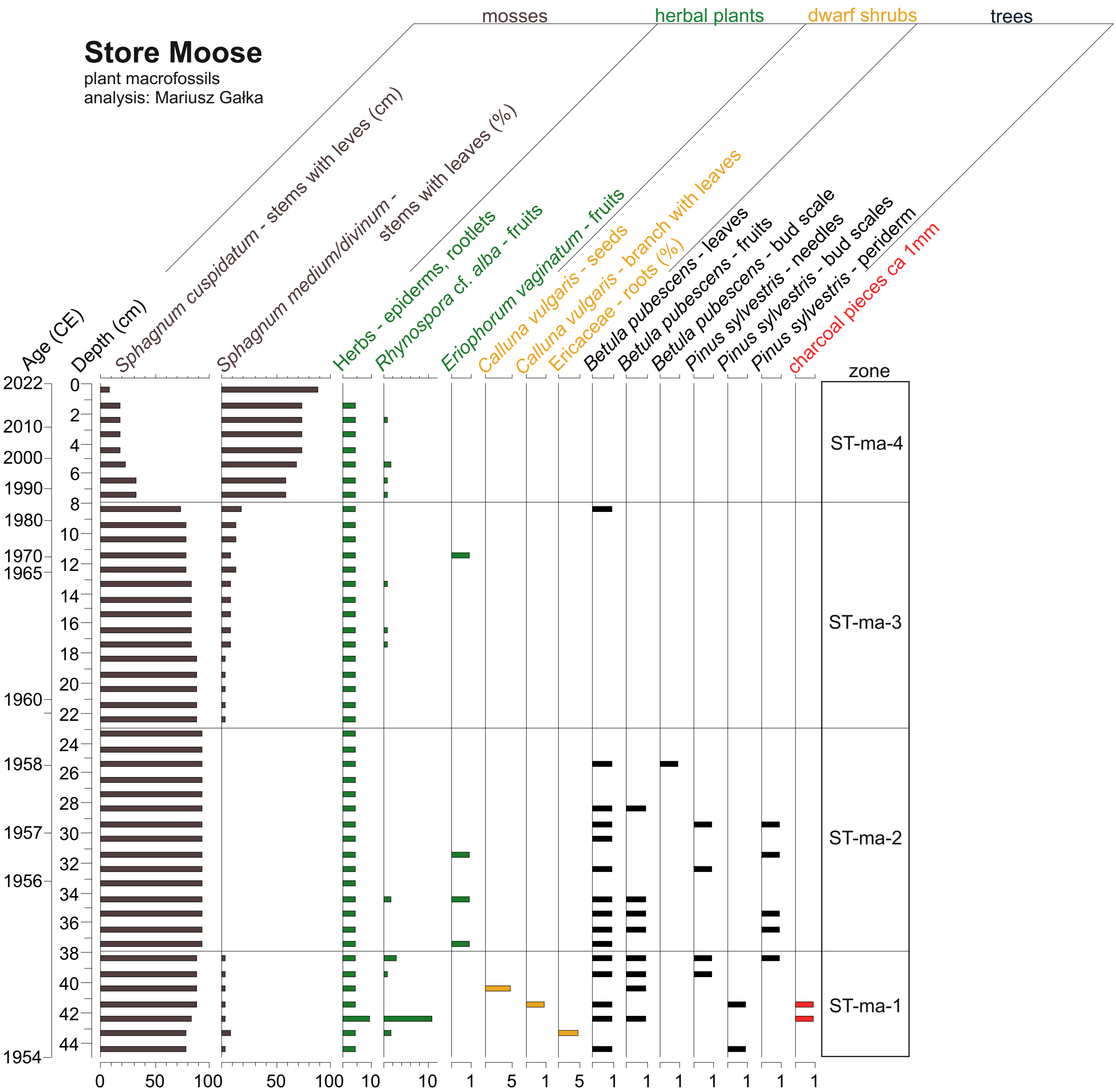

Store Moose
plant macrofossils
analysis: Mariusz Gałka
mosses
herbal plants
dwarf shrubs
trees
Age (CE)
Depth (cm)
Sphagnum cuspidatum - stems with leves (cm)
Sphagnum medium/divinum - stems with leaves (%)
Herbs - epiderms, rootlets
Rhynospora cf. alba - fruits
Eriophorum vaginatum - fruits
Calluna vulgaris - seeds
Calluna vulgaris - branch with leaves
Ericaceae - roots (%)
Betula pubescens - leaves
Betula pubescens - fruits
Betula pubescens - bud scale
Pinus sylvestris - needles
Pinus sylvestris - bud scales
Pinus sylvestris - periderm
charcoal pieces ca 1mm
zone
ST-ma-4
ST-ma-3
ST-ma-2
ST-ma-1
2022
2010
2000
1990
1980
1970
1965
1960
1958
1957
1956
1954
0
2
4
6
8
10
12
14
16
18
20
22
24
26
28
30
32
34
36
38
40
42
44
0 50 100 50 100 10 10 1 5 1 5 1 1 1 1 1 1 1

Suppl. Fig. 15

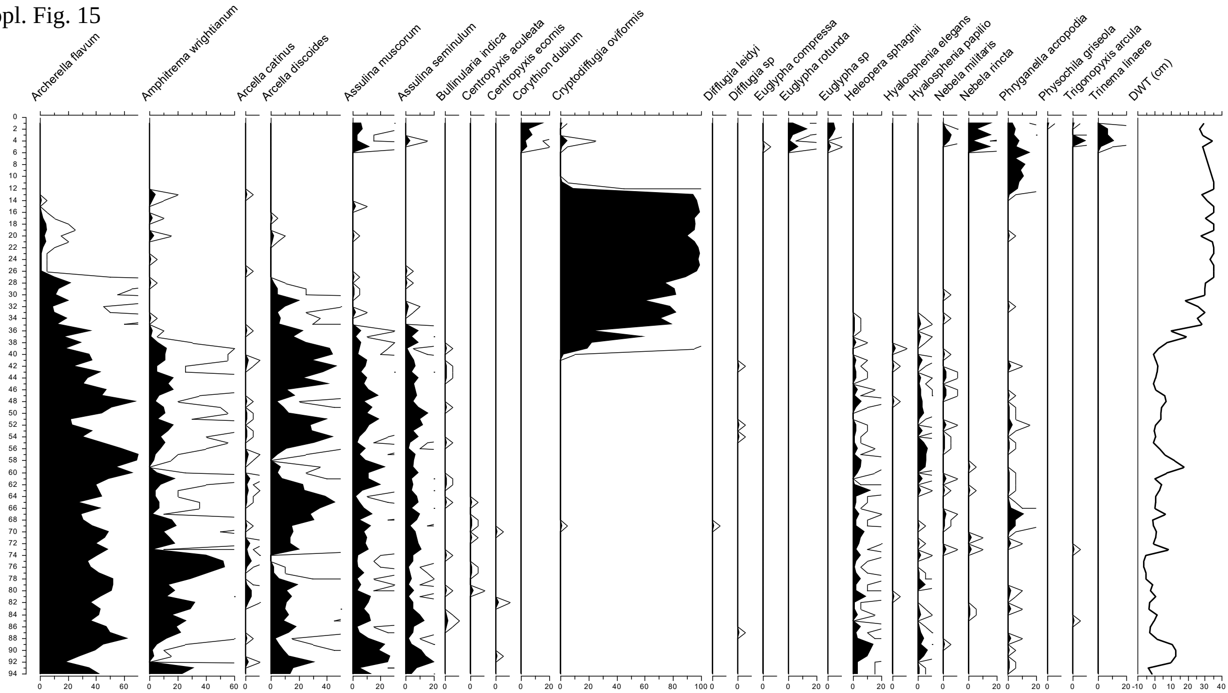
Archerella flavum
Amphitrema wrightianum
Arcella catinus
Arcella discoides
Assulina muscorum
Assulina seminulum
Bullinularia indica
Centropyxis aculeata
Centropyxis ecornis
Corythion dubium
Cryptodiffugia oviformis
Difflugia leidyi
Difflugia sp
Euglypha compressa
Euglypha rotunda
Euglypha sp
Heleopera sphagnii
Hyalosphenia elegans
Hyalosphenia papilio
Nebela militaris
Nebela rincta
Phryganella acropodia
Physochila griseola
Trigonopyxis arcula
Trinema linaere
DWT (cm)

Suppl. Fig. 16

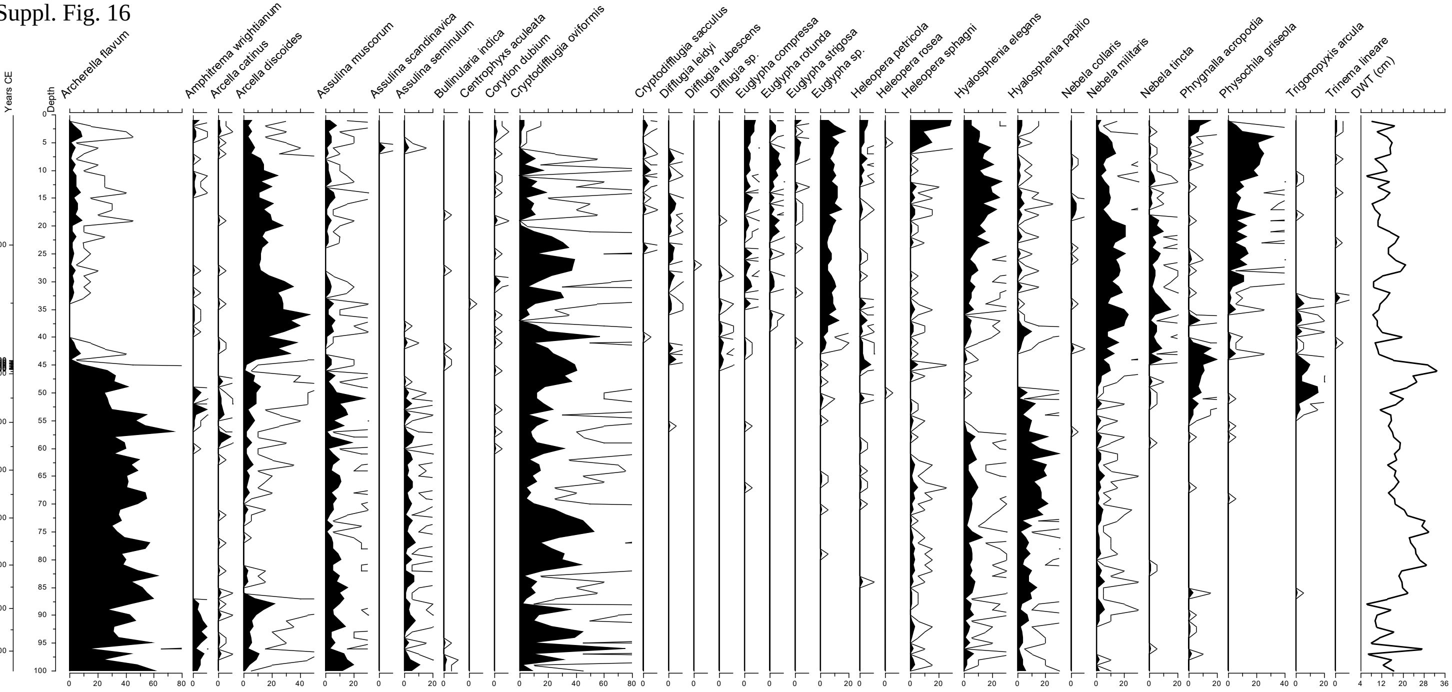
Years CE
Depth
Archerella flavum
Amphitrema wrightianum
Arcella catinus
Arcella discoides
Assulina muscorum
Assulina scandinavica
Assulina seminulum
Bullinularia indica
Centrophyxs aculeata
Corytion dubium
Cryptodifflugia oviformis
Cryptodifflugia sacculus
Difflugia leidyi
Difflugia rubescens
Difflugia sp.
Euglypha compressa
Euglypha rotunda
Euglypha strigosa
Euglypha sp.
Heleopera petricola
Heleopera rosea
Heleopera sphagni
Hyalosphenia elegans
Hyalosphenia papilio
Nebela collaris
Nebela militaris
Nebela tincta
Phrygnalla acropodia
Physochila griseola
Trigonopyxis arcula
Trinema lineare
DWT (cm)
2000
900
800
700
600
500
400
300
0
5
10
15
20
25
30
35
40
45
50
55
60
65
70
75
80
85
90
95
100

Suppl. Fig. 17

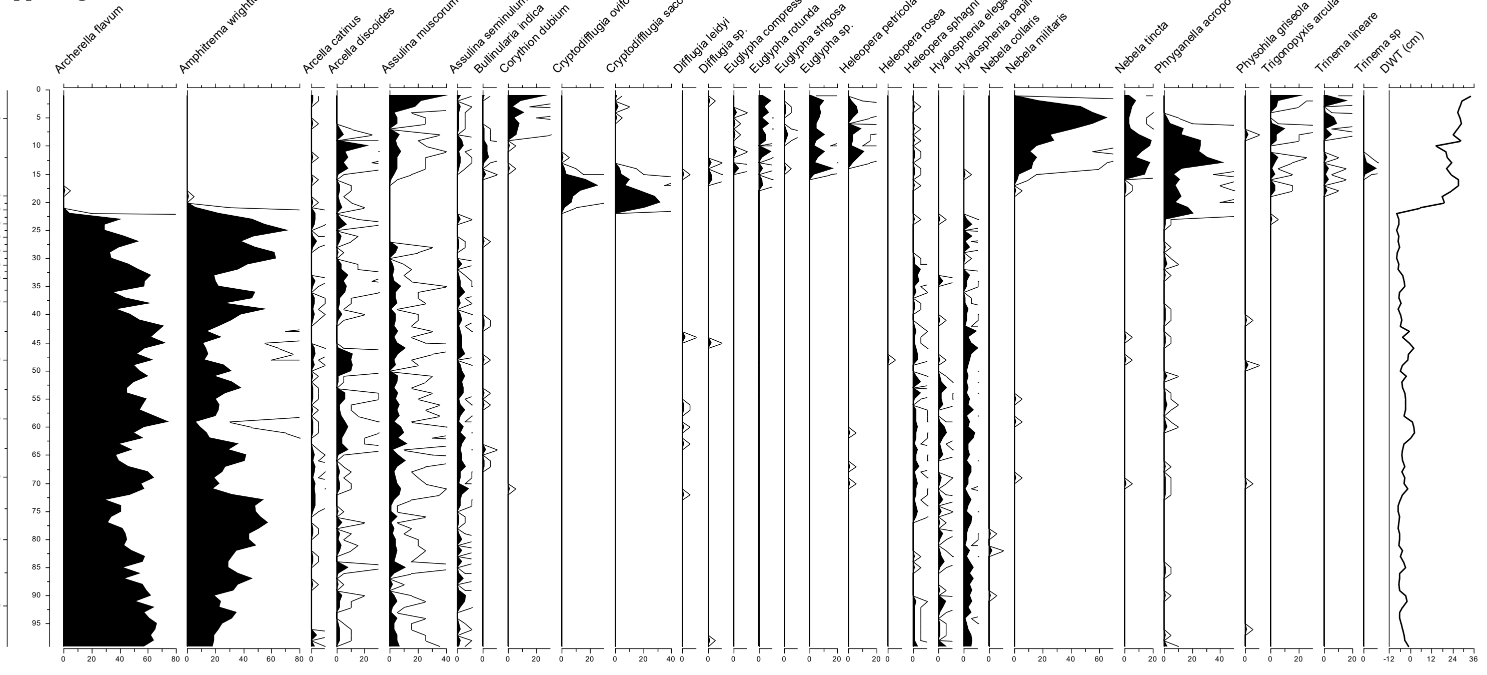
Archerella flavum
Amphitrema wrightianum
Arcella catinus
Arcella discoides
Assulina muscorum
Assulina seminulum
Bullinularia indica
Corythion dubium
Cryptodifflugia oviformis
Cryptodifflugia sacculus
Difflugia leidyi
Difflugia sp.
Euglypha compressa
Euglypha rotunda
Euglypha strigosa
Euglypha sp.
Heleopera petricola
Heleopera rosea
Heleopera sphagni
Hyalosphenia elegans
Hyalosphenia papilio
Nebela collaris
Nebela militaris
Nebela tincta
Phryganella acropodia
Physohila griseola
Trigonopyxis arcula
Trinema lineare
Trinema sp
DWT (cm)
2000
1900
1800
1700
1600
1500
1400
1300
1200
1100
1000
900
800
700
0
5
10
15
20
25
30
35
40
45
50
55
60
65
70
75
80
85
90
95
-12
12
24
36

Suppl. Fig. 18

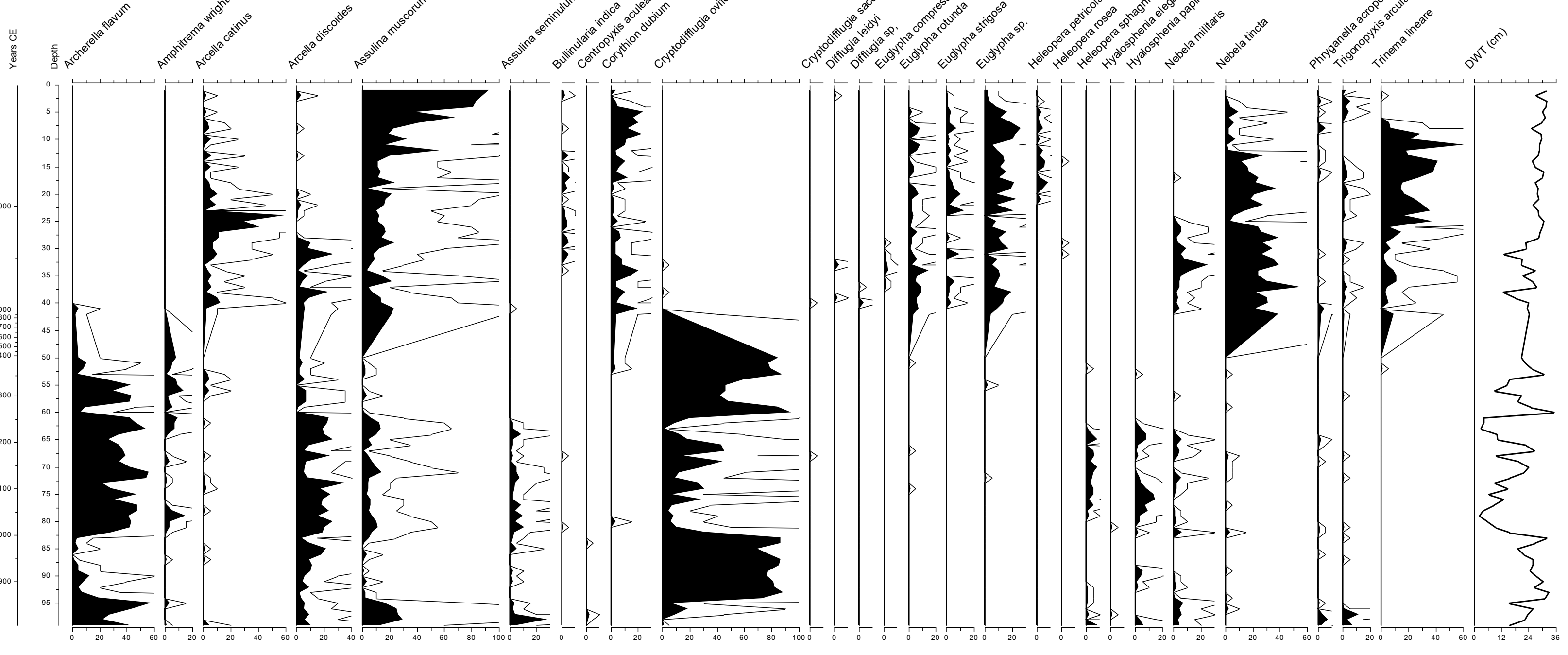

Years CE
Depth
Archerella flavum
Amphitrema wrightianum
Arcella catinus
Arcella discoides
Assulina muscorum
Assulina seminulum
Bullinularia indica
Centropyxis aculeata
Corythion dubium
Cryptodifflugia oviformis
Cryptodifflugia sacculus
Difflugia leidyi
Difflugia sp,
Euglypha compressa
Euglypha rotunda
Euglypha strigosa
Euglypha sp.
Heleopera petricola
Heleopera rosea
Heleopera sphagni
Hyalosphenia elegans
Hyalosphenia papilio
Nebela militaris
Nebela tincta
Phryganella acropodia
Trigonopyxis arcula
Trinema lineare
DWT (cm)
2000
1900
1800
1700
1600
1500
1400
1300
1200
1100
1000
900

Suppl. Fig. 19

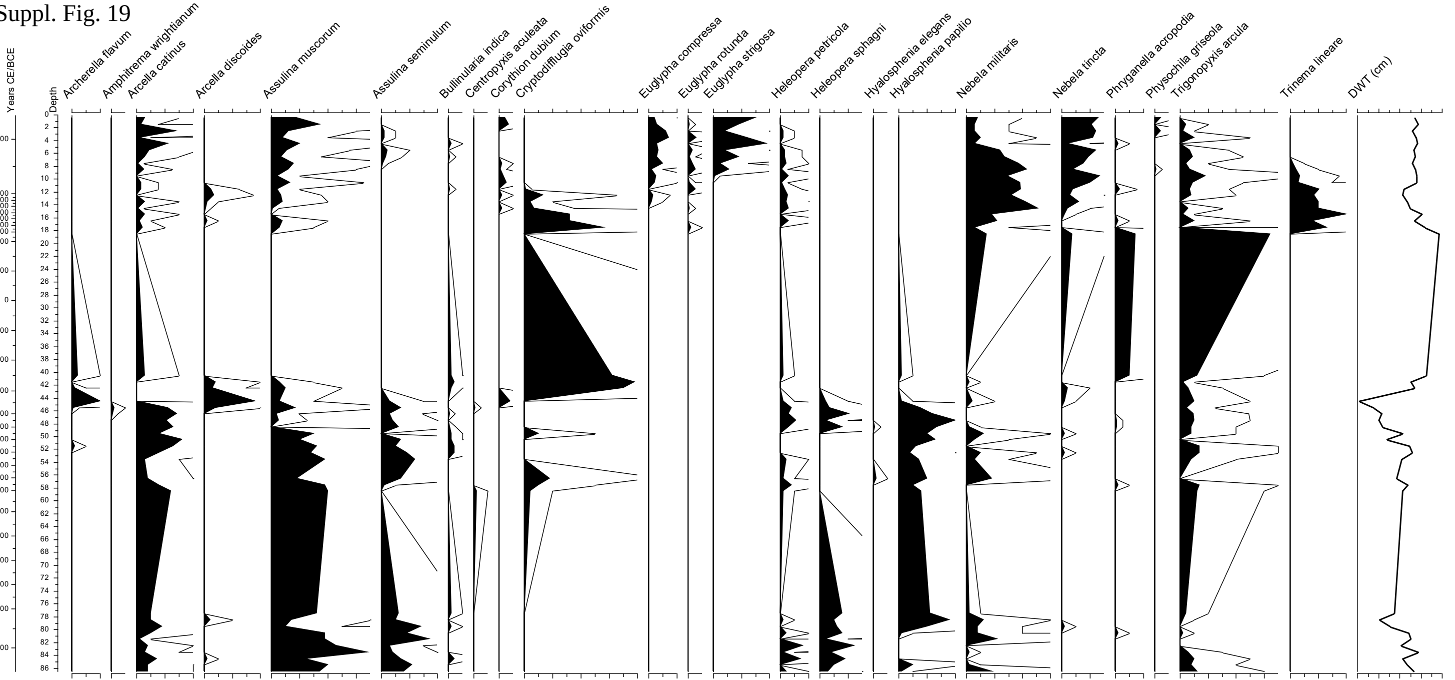
Years CE/BCE
Depth
Archerella flavum
Amphitrema wrightianum
Arcella catinus
Arcella discoides
Assulina muscorum
Assulina seminulum
Bullinularia indica
Centropyxis aculeata
Corythion dubium
Cryptodifflugia oviformis
Euglypha compressa
Euglypha rotunda
Euglypha strigosa
Heleopera petricola
Heleopera sphagni
Hyalosphenia elegans
Hyalosphenia papilio
Nebela militaris
Nebela tincta
Phryganella acropodia
Physochila griseola
Trigonopyxis arcula
Trinema lineare
DWT (cm)

Suppl. Fig. 20

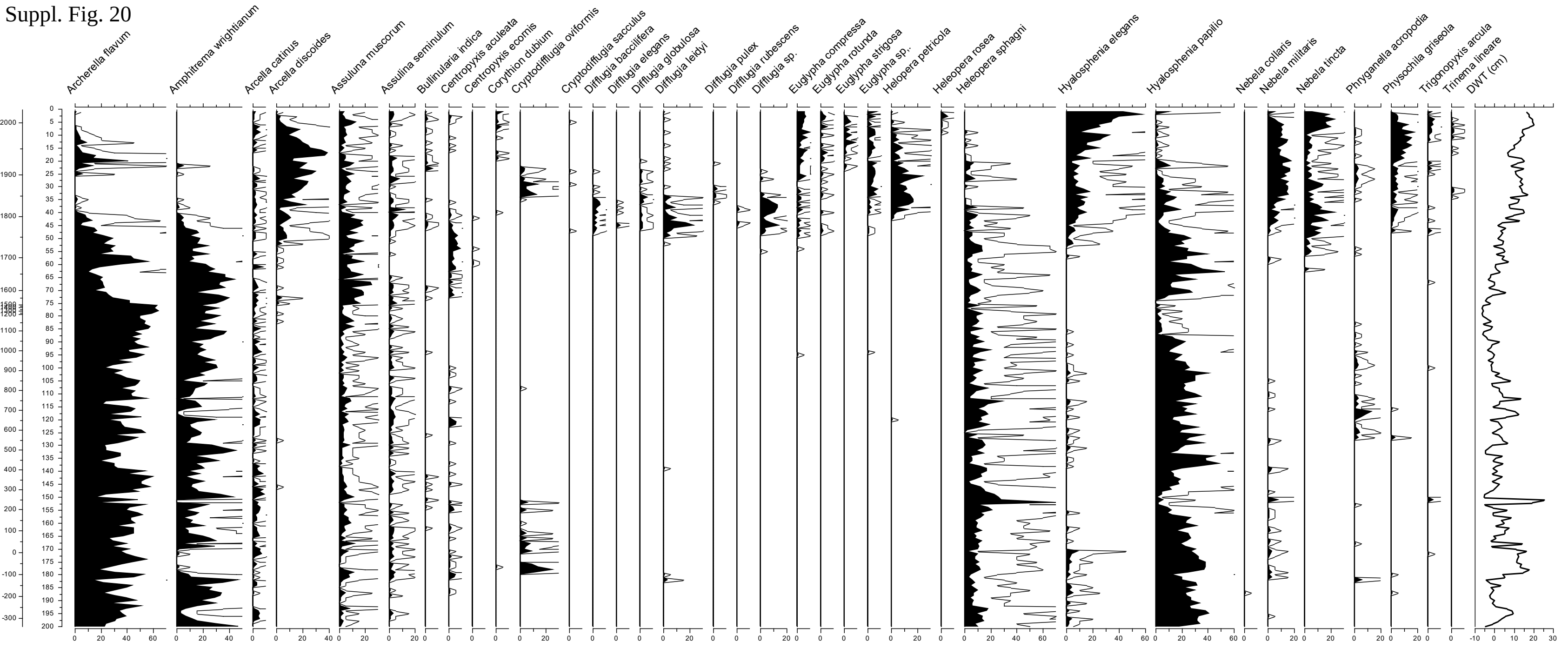
Archerella flavum
Amphitrema wrightianum
Arcella catinus
Arcella discoides
Assuluna muscorum
Assulina seminulum
Bullinularia indica
Centropyxis aculeata
Centropyxis ecornis
Corythion dubium
Cryptodifflugia oviformis
Cryptodifflugia sacculus
Difflugia bacillifera
Difflugia elegans
Difflugia globulosa
Difflugia leidyi
Difflugia pulex
Difflugia rubescens
Difflugia sp.
Euglypha compressa
Euglypha rotunda
Euglypha strigosa
Euglypha sp..
Helopera petricola
Heleopera rosea
Heleopera sphagni
Hyalosphenia elegans
Hyalosphenia papilio
Nebela collaris
Nebela militaris
Nebela tincta
Phryganella acropodia
Physochila griseola
Trigonopyxis arcula
Trinema lineare
DWT (cm)

Suppl. Fig. 21

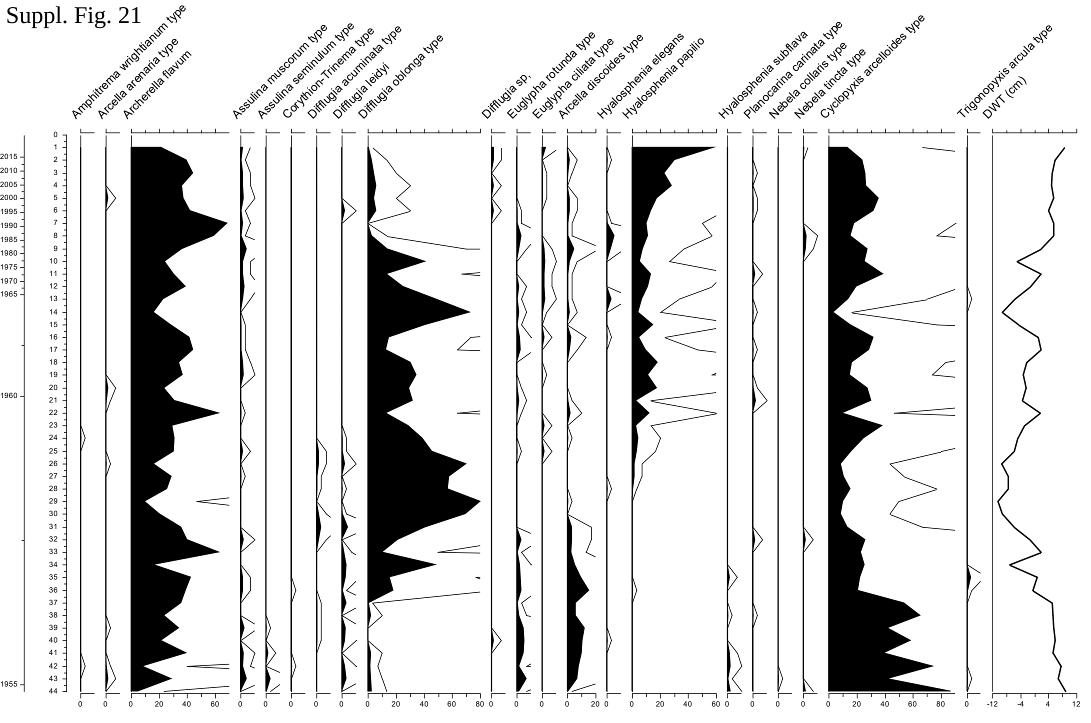

Amphitrema wrightianum type
Arcella arenaria type
Archerella flavum
Assulina muscorum type
Assulina seminulum type
Corythion-Trinema type
Difflugia acuminata type
Difflugia leidyi
Difflugia oblonga type
Difflugia sp,
Euglypha rotunda type
Euglypha ciliata type
Arcella discoides type
Hyalosphenia elegans
Hyalosphenia papilio
Hyalosphenia subflava
Planocarina carinata type
Nebela collaris type
Nebela tincta type
Cyclopyxis arcelloides type
Trigonopyxis arcula type
DWT (cm)

Tab. 1. Radiocarbon dates from various sites. Biodiversa project

Depth (cm)	Material	Nr. Lab.	C14 date	Age cal yr BP (95.4 %)	Age BCE/CE
	**POLAND, Bagno Kusowo**				
KUSREV 15-16	*Sphagnum* stems	Poz-152219	104.53 ± 0.34 pMmCodern	255-34	1696-1916
KUSREV 30-31	*Sphagnum* stems	Poz-152220	114.85 ± 0.36 pMmCodern	258-32	1692-1919
BK 35.5	*Sphagnum* stems	Poz-161646	135.07 ± 0.39 pMC	265-22	1686-1929
KUSREV 45-46	*Sphagnum* stems	Poz-152626	1265 ± 30 BP	1285-1079	666-871
KR1 59-60	*Sphagnum* stems	Poz-156100	1450 ± 30 BP	1379-1299	571-651
KR1 79-80	*Sphagnum* stems	Poz-156101	1530 ± 30 BP	1516-1347	434-603
KR1 98-99	*Sphagnum* stems	Poz-156103	1645 ± 30 BP	1686-1413	265-538
	**GERMANY, Vechtaer Moor**				
**Depth (cm)**	**Material**	**Nr. Lab.**	**C14 date**	**Age cal yr BP (95.4 %)**	**Age BC/AD**
DM1 14-15	*Sphagnum* stems	Poz-158121	105.93 ± 0.33 pMC modern	255-33	1695-1917
DM1 17-18	*Trichophorum cespitosum* fruits	Poz-158753	115.68 ± 0.36 pMC modern	295-31	1692-1919
DM1 35-36	*Sphagnum* stems, *Carex* sp. fruit, *Rhynospora alba* fruit	Poz-158122	900 ± 30 BP	909-732	1042-1219
DM1 73-74	*Sphagnum* stems	Poz-158123	1200 ± 30 BP	1244-1006	706-945
DM1 98-99	*Sphagnum* stems, *Oxycoccus palustris* leaves, *Andromeda polifolia* leaves	Poz-159301	1370 ± 30 BP	1346-1178	605-772
	**GERMANY, Amtsvenn Moor**				
AV1 7-8	*Baeothryon cespitosum* fruits	Poz-158751	130 ± 30 BP	275-8	1675-1942
AV1 15-16	*Sphagnum* stems	Poz-158752	1160 ± 30 BP	1178-973	773-978
AV1 27-28	*Sphagnum* stems	Poz-158118	1480 ± 30 BP	1400-1307	550-644
AV1 55-56	*Sphagnum* stems	Poz-158119	1670 ± 30 BP	1693-1420	275-531
AV1 93-95	*Sphagnum* stems	Poz-158107	2095 ± 30 BP	2146-1949	-197 - 1
	**Austria, Purgschachen Moor**				
PM1 16,5	*Sphagnum* stems	Poz-158108	103.01 ± 0.33 pMC modern	255-36	1696-1914
PM1 44,5	*Sphagnum* stems, *Oxyccocus palustris* leaves, *Rhynospora alba* fruit	Poz-158109	145 ± 30 BP	281-…	1669-…
PM1 65,5	*Sphagnum* stems	Poz-164571	170 ± 30 BP	291-…	1660-…
PM1 79,5	*Sphagnum* stems	Poz-158110	950 ± 30 BP	923-788	1028-1162
PM1 115,5	*Sphagnum* stems	Poz-158111	1320 ± 30 BP	1298-1176	652-775
PM1 156,5	*Pinus mugo* cone	Poz-158112	1880 ± 30 BP	1870-1714	81-236
PM1 198,5	*Sphagnum* stems	Poz-158120	2165 ± 30 BP	2306-2009	-357- 60
	**Austria, Pilchmeier Moor**				
PI1 11,5	*Pleurozium schreberi* stem with leaves + *Hylocomnium splenders* stems with leaves	Poz-159505	102.78 ± 0.32 pMC	254-37	1696-1914
PI1 19,5	*Sphagnum* stems	Poz-159506	1770 ± 30 BP	1728-1575	223-375
PI1 45,5	*Sphagnum* stems	Poz-159507	2450 ± 30 BP	2703-2361	-754- -412
PI1 65,5	*Sphagnum* stems	Poz-159508	4215 ± 35 BP	4854-4621	-2905- -2672
PI1 85,5	*Sphagnum* stems	Poz-159509	4415 ± 35 BP	5275-4865	-3326- -2916
	**Niederlands, Fochtelooër Veen**				

FV1 16,5	*Oxycoccus palustris leaves*	Poz-159510	101.47 ± 0.34 pMC modern	254-39	1697-1911
FV1 40,5	*Sphagnum* stems	Poz-159303	104.08 ± 0.35 pMC modern	255-35	1696-1916
FV1 47,5	*Sphagnum* stems and leaves, charcoal pieces	Poz-159512	570 ± 30 BP	644-526	1306-1424
FV1 62,5	*Sphagnum* stems	Poz-159513	820 ± 30 BP	775-678	1175-1273
FV1 98,5	*Sphagnum* stems	Poz-159514	1215 ± 30 BP	1262-1061	689-890
	**Stoore Moose, Sweden**				
SM13.5	*Sphagnum* stems	Poz-172186	100.67 ± 0.34 pMC	254-40	1697-1911
SM 21.5	*Sphagnum* stems	Poz-172187	103.16 ± 0.32 pMC	255-36	1696-1915
SM 33.5	*Sphagnum* stems	Poz-172188	104.44 ± 0.33 pMC	255-34	1696-1916
SM 43.5	*Sphagnum* stems	Poz-172189	108.12 ± 0.34 pMC	255-33	1695-1917
